# Janus kinase inhibitors in localized scleroderma: a systematic literature review

**DOI:** 10.55730/1300-0144.6000

**Published:** 2025-06-07

**Authors:** Seher ŞENER, Yusuf Ziya ŞENER, Ezgi Deniz BATU, Alper SARI, Ali AKDOĞAN

**Affiliations:** 1 Division of Pediatric Rheumatology, Department of Pediatrics, Adana City Training and Research Hospital, Adana, Turkiye; 2 Department of Cardiology, Erasmus University Medical Center, Rotterdam, Netherlands; 3 Division of Pediatric Rheumatology, Department of Pediatrics, Faculty of Medicine, Hacettepe University, Ankara, Turkiye; 4 Division of Rheumatology, Department of Internal Medicine, Ankara Etlik State Research and Training Hospital, Ankara, Turkiye; 5 Division of Rheumatology, Department of Internal Medicine, Faculty of Medicine, Hacettepe University, Ankara, Turkiye

**Keywords:** Biologic drugs, localized scleroderma, Janus kinase inhibitors, side effects, treatment

## Abstract

**Background/aim:**

Reports on the use of Janus kinase (JAK) inhibitors in the treatment of localized scleroderma are increasing in the literature. In this review, we examined the published studies regarding the use of JAK inhibitors in patients with localized scleroderma.

**Materials and methods:**

We searched MEDLINE and Scopus for articles on patients with localized scleroderma treated with JAK inhibitors. The search included articles from the inception of these databases through August 1^st^, 2024.

**Results:**

Our literature search showed 11 articles describing 17 patients with localized scleroderma treated with JAK inhibitors. Generalized morphea (47.1%) was the most common type of localized scleroderma in patients treated with JAK inhibitors. The most frequently used JAK inhibitor was tofacitinib (64.7%). In some cases, baricitinib (17.6%) and ruxolitinib (17.6%) were also preferred. All JAK inhibitors were mainly preferred for the treatment of resistant/progressive skin disease in these patients (60.7%). The improvement rate associated with JAK inhibitors was 88.2%. Relapse occurred in 33.3% of patients treated with JAK inhibitors. Side effects were reported in 18.2% of patients: one patient was diagnosed to have diffuse large B-cell lymphoma (n = 1) while on tofacitinib.

**Conclusion:**

JAK inhibitors could be considered as a therapeutic option, especially in patients with refractory localized scleroderma, but more extensive clinical trials are needed to clarify questions regarding their efficacy and safety data.

## 1. Introduction

Localized scleroderma, the most common type of morphea, is a condition characterized by hardened, thickened patches of skin [1]. Unlike systemic sclerosis, it does not affect the internal organs [2]. The exact cause of localized scleroderma is unknown, but it is thought to involve an overactive immune response that leads to excessive collagen production [2].

Localized scleroderma requires a multifaceted treatment approach to manage symptoms, reduce skin changes, and prevent complications [3]. The choice of treatment depends on the severity and extent of the disease [4]. Various conventional or biologic disease-modifying anti-rheumatic drugs (DMARDs) are used with topical or systemic corticosteroids [3].

Janus kinase (JAK) inhibitors work by interfering with the signaling pathways that promote inflammation and fibrosis [5]. In localized scleroderma, where excessive collagen deposition leads to skin thickening, JAK inhibitors may help reduce this fibrotic process [6]. Early studies and case reports suggest that JAK inhibitors may be beneficial for patients with localized scleroderma, particularly those who do not respond well to conventional therapies [7–9].

In this systematic review, we aimed to analyze published data on the use of JAK inhibitors in the treatment of localized scleroderma.

## 2. Methods and search strategy

We conducted a systematic search through PubMed/MEDLINE and Scopus using the following keywords: “localized scleroderma”, “Janus kinase inhibitor”, “JAK inhibitor”, “tofacitinib”, “baricitinib”, “ruxolitinib”, “upadacitinib”, “filgotinib”, “peficitinib”, “decernotinib”, according to the PRISMA guidelines [10]. The search covered articles from the inception of these databases to August 1^st^, 2024, and was limited to English-language articles focusing on human studies. We included case reports/series, original research articles, editorials, and review articles that involved patients with localized scleroderma treated with JAK inhibitors. Only articles containing relevant data were considered for the final analysis. Two authors (YZS and SS) independently conducted the literature search, removing irrelevant literature, eliminating duplicates, and screening titles, abstracts, and full texts. Data extraction from the included studies was also performed independently by the authors, with any disagreements resolved through consensus.

## 3. Results of the systematic review

**Figure 1:**
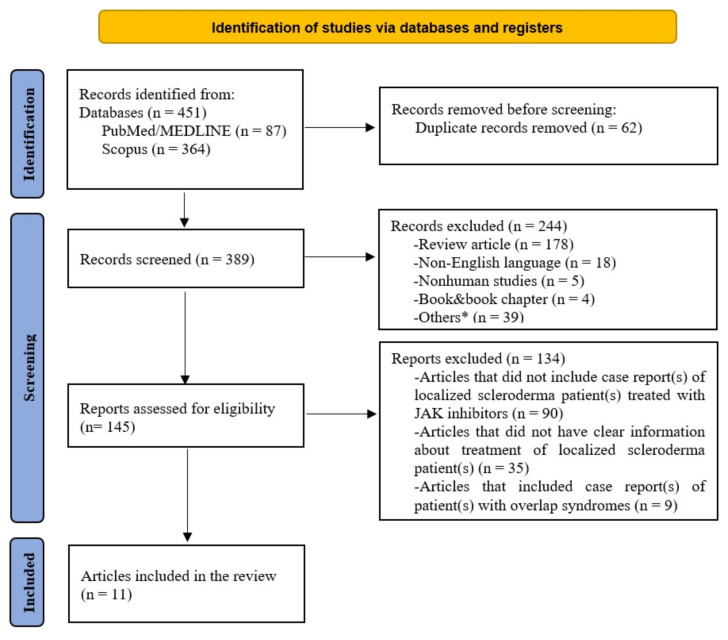
The PRISMA flow diagram of literature screening regarding Janus kinase inhibitor use in patients with localized scleroderma. *Others: guideline, letter, editorial, conference paper, short survey, comment, note JAK, Janus kinase

Our literature search identified 11 articles describing 17 patients with localized scleroderma treated with JAK inhibitors [7–9, 11–18] (Figure 1). Table 1 summarized the characteristics of patients with localized scleroderma treated with JAK inhibitors. Detailed data for all patients were shown in Supplementary Table 1.

The median (min-max) age of localized scleroderma patients treated with JAK inhibitors in the literature was 53 (4–84) years (female/male = 9/8) (Table 1). The most common type of localized scleroderma in the patients was generalized morphea (47.1%), and joint involvement (mostly arthritis, 75%) was the most frequent extra-cutaneous involvement.

The most commonly used JAK inhibitor in patients with localized scleroderma was tofacitinib (64.7%) (Table 1). In some cases, baricitinib (17.6%) and ruxolitinib (17.6%) were also preferred. At least one immunosuppressive agent was used previously and/or concomitantly in most patients receiving JAK inhibitors. Corticosteroids (70.6%) and methotrexate (MTX, 70.6%) were the most frequently used other immunosuppressive drugs.

**Figure 2:**
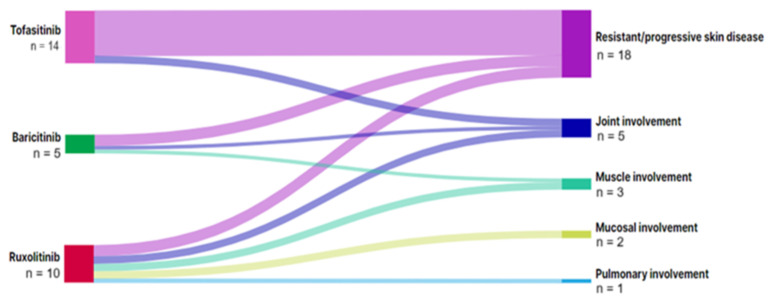
The diagram representing the indications for Janus kinase inhibitors in localized scleroderma. ^a^n represents the number of treatments

Out of a total of 28 indications for starting a JAK inhibitor, the most common one was resistant/progressive skin disease (17/28, 60.7%) (Table 2). All JAK inhibitors were mainly preferred for this reason in patients with localized scleroderma (Figure 2).

Detailed information on JAK inhibitor dosage and treatment duration was available in most of the included studies. For tofacitinib, the reported daily dose ranged from 5 to 10 mg, with a median treatment duration of 6 (4–11) months. Baricitinib was used at doses of 2 to 4 mg/day for a median duration of 6 (6–12) months. Ruxolitinib was administered at 20 mg/day with a median duration of 28 (18–28) months.

Improvement was achieved in 15/17 patients (88.2%). Relapse occurred in 33.3% of the patients on JAK inhibitors. Side effects were reported in two of 11 patients (18.2%) as diffuse large B-cell lymphoma (n = 1) possibly associated with tofacitinib and anxiety (n = 1) with ruxolitinib.

## 4. Discussion

This systematic review showed that JAK inhibitors are used in localized scleroderma patients, especially for refractory/progressive skin disease, and the most commonly used JAK inhibitor was tofacitinib. Although the limited number of patients makes it challenging to comment on safety, JAK inhibitors seem to be effective in localized scleroderma treatment.

The pathogenesis of localized scleroderma is complex and multifactorial, with transforming growth factor-beta (TGF-β) playing a central role in driving fibrosis through the activation of fibroblasts and the overproduction of extracellular matrix components [19, 20]. JAK operates downstream of TGF-beta in profibrotic signaling, and activation of the JAK/STAT pathway has been shown to result in fibrosis [19]. JAK phosphorylates signal transducers and activators of transcription (STAT) proteins, initiating the transcription of target genes, including those that are profibrotic and proinflammatory [21]. JAK inhibitors have been shown to inhibit TGF-β mediated effects in skin sclerosis in both in vitro and animal models [13, 22].

Current treatment recommendations for localized scleroderma are limited [23]. In addition, these are not disease-specific treatments and long-term use of immunosuppressive agents is associated with side effects [23]. Therefore, there is an unmet need for both understanding the pathogenesis of localized scleroderma and identifying targeted therapies.

Corticosteroids alone do not provide long-term benefits and are often used in combination with MTX and other treatments in localized scleroderma treatment [23]. Methotrexate is recommended and widely used as a first-line treatment for active, moderate, and severe disease [24, 25]. Although there is only one randomized, double-blind, placebo-controlled trial (RCT) of MTX [26], there are many case reports supporting its efficacy and safety [27, 28]. It was found that 67% of patients in the MTX group achieved a clinical response in this RCT [26]. In the remaining 30% of patients, no improvement was observed, and the need for further treatment in this group of patients comes to the fore.

The Childhood Arthritis and Rheumatology Research Alliance (CARRA) localized scleroderma group has developed three main treatment plans: MTX monotherapy, MTX with intravenous glucocorticoids, or MTX with oral glucocorticoids [29]. If disease activity does not improve after 6 months with these treatment plans, or if activity worsens after 3–4 months, further treatment is required [23]. Additional treatment includes a course of intravenous corticosteroids and/or increasing the dose of oral corticosteroids or adding mycophenolate mofetil (MMF) instead of or in addition to MTX [30]. Apart from these regimens, there are several case reports demonstrating the efficacy of other conventional DMARDs like hydroxychloroquine and cyclosporine A and biologic DMARDs such as abatacept, anti-interleukin-6 (tocilizumab), and anti-tumor necrosis factor-alpha as second-line treatments [31–34]. However, despite all these treatments, there are still cases of localized scleroderma that remain refractory [23].

Recently, there have been promising reports in the literature regarding the use of JAK inhibitors in localized scleroderma [35]. JAK inhibitors are highly effective, particularly in case reports of adult localized scleroderma [35]. Our literature review demonstrated that JAK inhibitors, especially tofacitinib, are effective in patients with resistant/progressive skin disease and localized scleroderma who were already resistant to multiple immunosuppressive agents. Therefore, JAK inhibitors could be considered an effective treatment option in patients with localized scleroderma, especially in refractory cases. Furthermore, improvements in a patient’s pulmonary involvement and muscle involvement were in line with the reports about the effectiveness of JAK inhibitors in inflammatory myositis and interstitial lung disease.

There are concerning potential side effects of JAK inhibitors, including hematologic, hepatic, and renal abnormalities, hyperlipidemia, thromboembolism, increased risk of viral and bacterial infections, and malignancies [19]. Upper respiratory tract infections are one of the most common side effects of treatment with the JAK inhibitors [36], but this side effect was not mentioned in any patients with localized scleroderma in the published studies. Except for one case of lymphoma after using JAK inhibitors in the literature [16], studies conducted to date have generally shown that JAK inhibitors are well tolerated and do not cause significant side effects in patients with localized scleroderma.

This systematic review has several limitations. A key concern is the potential for publication bias, which may skew the overall conclusions toward favorable outcomes, as unsuccessful or negative results are less likely to be reported. Another major limitation is the small sample size and reliance primarily on case reports and small case series, which not only reduces the generalizability of the findings but also introduces inherent biases due to selective reporting, lack of control groups, and the absence of standardized data collection, thereby precluding any meaningful meta-analytic synthesis. Furthermore, the included studies show considerable heterogeneity in study designs, treatment protocols, and outcome measures. While some studies relied on physician global assessment or imaging findings, others used subjective improvement reports or reduction in lesion size. This lack of standardized assessment tools hampers direct comparison across studies and introduces subjectivity into the outcome evaluation. Additionally, the majority of studies did not consistently report JAK inhibitor dosages or treatment durations, limiting conclusions regarding optimal therapeutic regimens. Taken together, these factors necessitate cautious interpretation of the current findings and highlight the need for well-designed prospective studies.

## 5. Conclusion

Data regarding the therapeutic effects and safety data of JAK inhibitors, especially in patients with localized scleroderma, are limited to case reports and small case series. Given the emerging but preliminary evidence, JAK inhibitors may be cautiously considered in patients with refractory localized scleroderma who fail to respond to conventional therapy. However, careful monitoring for adverse events is essential, and treatment decisions should be individualized until further evidence becomes available. More extensive clinical trials are needed to establish their efficacy and safety in the use of JAK inhibitors for localized scleroderma. However, early results are promising, suggesting that these medications could offer a new therapeutic option for patients with this challenging condition.

## Supplementary Information



## Figures and Tables

**Table 1:** Characteristics of patients with localized scleroderma treated with Janus kinase inhibitors in the literature.

Number of patients, n	17

Age at diagnosis, years, median (min-max)	53 (4–84)

Pediatric/adult patients, n	6/11

Sex, female, n (%)	9 (52.9)

Type of localized scleroderma, n (%)	
Generalized morphea	8 (47.1)
Pansclerotic morphea	4 (23.6)
Linear scleroderma	3 (17.6)
Morphea	2 (11.8)

Extra-cutaneous symptoms, n (%)	
Joint involvement	9/12 (75)
Muscle involvement (myositis or isolated muscle involvement)	4/12 (33.3)
Constitutional symptoms	3/12 (25)
Mucosal involvement	2/12 (15.4)
Others*	4/12 (33.3)

Laboratory findings, n (%)	
ANA positivity (≥1/160)	1/6 (16.7)
Anti-Scl-70 positivity	0/6 (0)

JAK inhibitors, n (%)	
Tofacitinib	11 (64.7)
Baricitinib	3 (17.6)
Ruxolitinib	3 (17.6)

Other treatments, n (%)	
Corticosteroid	12 (70.6)
Methotrexate	12 (70.6)
Topical therapy	5 (41.7)
Phototherapy	5 (41.7)
Photopheresis	4 (23.5)
Mycophenolate mofetil	4 (23.5)
IVIG	4 (23.5)
Hydroxychloroquine	3 (17.6)
Cyclosporine A	2 (11.8)
Cyclophosphamide	2 (11.8)
Infliximab	2 (11.8)
Etanercept	2 (11.8)
Tocilizumab	2 (11.8)
Bosentan	2 (11.8)
NSAID	2 (11.8)
Others**	8 (47.1)

Disease duration, years, median (min-max)	2.4 (0.5–9)

Outcome, n (%)	
Improvement	15 (88.2)
No improvement	2 (11.8)

ANA, antinuclear antibody; IVIG, intravenosus immunoglobulin; NSAID, nonsteroidal antiinflammatory drugs; JAK, Janus kinase

* pulmonary arterial hypertension (n = 1), lymphadenopathy (n = 1), hepatosplenomegaly (n = 1), lipodystrophy (n = 1)

** rituximab (n = 1), thalidomide (n = 1), apremilast (n = 1), imatinib (n = 1), antitymocyte globulin (n = 1), autologous stem cell transplantation (n = 1), alprostadil (n = 1), minocycline (n = 1), glycyrrhizin (n = 1)

**Table 2:** Treatment reasons and responses in patients with localized scleroderma treated with Janus kinase inhibitors.

JAK inhibitors	Indication for JAK inhibitors^a^	Response to JAK inhibitors^a^	Adverse event^a^	Relapse under JAK inhibitors^a^
Tofacitinib (n = 11s)	Resistant/progressive skin disease (n = 11)	Improvement (n = 9) No improvement (n = 2)	Diffuse large B-cell lymphoma (possible, n = 1)	1/3^b^ (33.3%)
	Joint involvement (n = 2)	Improvement (n = 2)		
Baricitinib (n = 3)	Resistant/progressive skin disease (n = 3)	Improvement (n = 3)		
	Joint involvement (n = 1)	Improvement (n = 1)		
	Muscle involvement (n = 1)	Improvement (n = 1)		
Ruxolitinib (n = 3)	Resistant/progressive skin disease (n = 3)	Improvement (n = 3)	Anxiety (n = 1)	
	Joint involvement (n = 2)	Improvement (n = 2)		
	Muscle involvement (n = 2)	Improvement (n = 2)		
	Mucosal involvement (n = 2)	Improvement (n = 2)		
	Pulmonary involvement (n = 1)	Improvement (n = 1)		

JAK, Janus kinase

a n represents the number of treatments

b Number of patients with reported relapse status
