# Supplementary material for: Janus kinase inhibitors in localized scleroderma: a systematic literature review
Source: Turk J Med Sci. 2025 Jun 7;55(3):533–9. doi: 10.55730/1300-0144.6000 (PMC12270291; doi:10.55730/1300-0144.6000)
Supplement: Supplementary file 1 [file tjmed-55-03-533_supplementarytable.docx]

**Supplementary Table 1.** The characteristics of patients with localized scleroderma treated with Janus kinase inhibitors

| **First author, year (ref. no.)** | **No. of patients** | **Age at diagnosis (years)** | **Sex** | **Type of localized scleroderma** | **Extra-cutaneous involvements** | **Laboratory findings** | **Treatments** | **Disease duration (years)** | **Relapse undue JAK inhibitor** | **Reasons for initiating JAK inhibitor** | **Responses to JAK inhibitor** | **Adverse events** |
| --- | --- | --- | --- | --- | --- | --- | --- | --- | --- | --- | --- | --- |
| Kim, 2018 (15) | 2 | 53, 66 | 2F | Generalized deep morphea (n=2) | Joint involvement (n=2) | ANA (-) (n=1), anti- Scl-70 (-) (n=1) | CS (n=2), MTX (n=2), photopheresis (n=1), tofacitinib (n=2) | NI (n=2) | NI (n=2) | Resistant/progressive skin disease (n=2), joint involvement (n=2) | Improvement for all reasons (n=2) | None (n=2) |
| Soh, 2019 (19) | 1 | 5 | M | Disabling pansclerotic morphea | Constitutional symptoms, joint involvement, LAP, HSM, lipodystrophy | ANA (-) | NSAID, CS, MTX, IVIG, HCQ, MMF, RTX, TOC, bosentan, ruxolitinib | 1.2 | NI | Resistant/progressive skin disease | Improvement | NI |
| Scheinberg, 2020 (18) | 1 | 59 | M | Morphea | NI | NI | CS, MTX, tofacitinib | NI | NI | Resistant/progressive skin disease | Improvement | NI |
| Damsky, 2020 (14) | 4 | 71 (54-84)* | 3F/1M | Generalized morphea (n=4) | NI (n=4) | NI (n=4) | CS (n=3), MTX (n=3), MMF (n=1), phototherapy (n=1), photopheresis (n=1), topical treatment (n=1), tofacitinib (n=3), baricitinib (n=1) | 4.1 (2.8-4.8)* | NI (n=4) | Resistant/progressive skin disease (n=4) | Improvement (n=4) | None (n=4) |
| Koschitzky, 2021 (16) | 1 | 68 | F | Morphea | Joint involvement | NI | MTX, apremilast, phototherapy, topical treatment, tofacitinib | 1.9 | 1 | Resistant/progressive skin disease | No improvement | None |
| Chebli de Abreu, 2022 (13) | 1 | 58 | M | Pansclerotic morphea | Constitutional symptoms, joint, and muscle involvement | ANA (-), anti- Scl-70 (-) | CS, CsA, CYC, MMF, IVIG, topical treatment, TOC, ETN, tofacitinib | 0.5 | NI | Resistant/progressive skin disease | No improvement | NI |
| Liu, 2022 (17) | 1 | 50 | M | Generalized morphea | Joint involvement | ANA (+), anti- Scl-70 (-) | CS, thalidomide, HCQ, alprostadil, topical treatment, tofacitinib | 0.8 | NI | Resistant/progressive skin disease | Improvement | Diffuse large B-cell lymphoma (relationship with tofacitinib not certain) |
| Labrandero Hoyos, 2023 (8) | 1 | 68 | F | Generalized morphea | None | NI | CS, MTX, MMF, IFX, photopheresis, baricitinib | NC | NI | Resistant/progressive skin disease | Improvement | None |
| Tang, 2023 (9) | 2 | 6, 13 | 1F/1M | Linear scleroderma (n=2) | None (n=2) | ANA (-) (n=1), NC (n=1) | MTX (n=1), HCQ (n=1), glycyrrhizin (n=1), phototherapy (n=2), topical treatment (n=1), tofacitinib (n=2) | NI (n=2) | NC (n=2) | Resistant/progressive skin disease (n=2) | Improvement (n=2) | None (n=1), NI (n=1) |
| Baghdassarian, 2023 (12) | 2 | 5, 16 | 2M | Disabling pansclerotic morphea (n=2) | Mucosal (n=2), joint (n=2), muscle (n=2), constitutional symptom (n=1), PAH (n=1) | NI (n=2) | CS (n=2), NSAID (n=1), MTX (n=2), MMF (n=1), CsA (n=1), CYC (n=1), IVIG (n=2), phototherapy (n=1), minocycline (n=1), imatinib (n=1), bosentan (n=1), ATG (n=1), ETN (n=1), IFX (n=1), ASCT (n=1), ruxolitinib (n=2) | 1.5, 9 | None (n=2) | Resistant/progressive skin disease (n=2), mucosal (n=2), joint (n=2), muscle (n=2) involvement, PAH (n =1) | Improvement for all reasons (n=2) | None (n=1), anxiety (n=1) |
| Zou, 2024 (10) | 1 | 4 | F | Linear scleroderma | Joint and muscle involvement | ANA (-) | Baricitinib | NI | NI | Resistant/progressive skin disease, joint and muscle involvement | Improvement for all reasons | None |

*ANA, antinuclear antibody; ASCT, autologous stem cell transplantation; ATG, anti-thymocyte globulin; CS, corticosteroid; CsA, cyclosporine; CYC, cyclophosphamide; ETN, etanercept; F, female; HCQ, hydroxychloroquine; HSM, hepatosplenomegaly; IFX, infliximab; IVIG, intravenous immunoglobulin;* *LAP, lymphadenopathy; M, male; MMF, mycophenolate mofetil; MTX, methotrexate; NC, not clear; NI, not indicated; NSAID, nonsteroidal anti-inflammatory drugs; JAK, Janus kinase; PAH, pulmonary arterial hypertension; RTX, rituximab; TOC, tocilizumab*

**median (minimum-maximum)*
